# Book Reviews

**Published:** 1799-03

**Authors:** 


### THE VETERINARY ART.

*Le parfait Bouvier,* &c. *The complete Herdſman.* By Boutrolle. 12mo. *Paris.* Huzard.

This is a new edition, with conſiderable improvements, of a very uſeful and well written work on the management of cattle; their age; their maladies; and the beſt methods of cure. To the preſent edition are added, two ſmall treatiſes, of conſiderable merit, on ſheep and hogs, together with ſome new medicines for horſes.

*Inſtructions ſur les moyen les plus propres à aſſurer la propagation des bêtes à laine de race d'Eſpagne, &c.* "*Directions reſpecting the moſt proper Means of promoting the Propagation of the Sheep affording Spaniſh Wool, and the Preſervation of their Breed in its Purity,*" publiſhed by the Council of Agriculture, and edited by Gilbert, 8vo. (30 ſols) *Paris.* Bureau du Nouvelliſte.

*Inſtruction*

*Instruction sur le claveau des moutons.* "Advice on the Rot in Sheep." 8vo. (20 sols) *Paris.* Ibid.

These two practical works we have placed together; as they deserve to be jointly recommended to the attention of all who are interested in improving this valuable branch of rural œconomy. The British farmer and grazier could not be treated with a more useful and acceptable present, than the translation of these little volumes into the English language.

